# Implications for neutrophils in cardiac arrhythmias

**DOI:** 10.1152/ajpheart.00590.2023

**Published:** 2023-12-15

**Authors:** Niklas Hegemann, Lukas Barth, Yannic Döring, Niels Voigt, Jana Grune

**Affiliations:** ^1^Department of Cardiothoracic and Vascular Surgery, Deutsches Herzzentrum der Charité (DHZC), Berlin, Germany; ^2^Charité-Universitätsmedizin Berlin, corporate member of Freie Universität Berlin and Humboldt-Universität zu Berlin, Berlin, Germany; ^3^German Centre for Cardiovascular Research (DZHK), Berlin, Germany; ^4^Institute of Pharmacology and Toxicology, University Medical Center Göttingen, Georg August University Göttingen, Göttingen, Germany; ^5^German Centre for Cardiovascular Research (DZHK), Göttingen, Germany; ^6^Cluster of Excellence “Multiscale Bioimaging: from Molecular Machines to Networks of Excitable Cells” (MBExC), University of Göttingen, Göttingen, Germany

**Keywords:** arrhythmia, immune system, mechanisms, molecular, neutrophils

## Abstract

Cardiac arrhythmias commonly occur as a result of aberrant electrical impulse formation or conduction in the myocardium. Frequently discussed triggers include underlying heart diseases such as myocardial ischemia, electrolyte imbalances, or genetic anomalies of ion channels involved in the tightly regulated cardiac action potential. Recently, the role of innate immune cells in the onset of arrhythmic events has been highlighted in numerous studies, correlating leukocyte expansion in the myocardium to increased arrhythmic burden. Here, we aim to call attention to the role of neutrophils in the pathogenesis of cardiac arrhythmias and their expansion during myocardial ischemia and infectious disease manifestation. In addition, we will elucidate molecular mechanisms associated with neutrophil activation and discuss their involvement as direct mediators of arrhythmogenicity.

## ELECTROIMMUNOLOGY: A NEW PHYSIOLOGICAL CONCEPT

Cardiac arrhythmias represent a major burden on healthcare systems and societies worldwide. This is, at least in part, due to severe potential consequences such as sudden cardiac death and stroke (1, 2), indicating a high public demand for the development of safe and effective treatment options. This, however, requires an in-depth understanding of the mechanisms underlying cardiac arrhythmias. The electrophysiological mechanisms leading to ventricular and atrial rhythm disorders have been extensively studied in the past, but the societal burden of arrhythmia remains significant (3–5). Hence, expanding our understanding of arrhythmia pathogenesis beyond classical electrophysiological concepts may help to develop new approaches for prevention and targeted treatment options.

In the light of recent scientific evidence, we pick up on the novel concept of electroimmunology, investigating the role of immune cells in electrical conduction of the healthy and diseased heart (6). More specifically, this review will focus largely on the role of leukocyte populations, mainly neutrophils, monocytes, and macrophages. Conditions that increase arrhythmia risk such as ischemia or infections are associated with substantial changes in myocardial leukocyte counts and phenotypes (7). Previously, this cardiac inflammation was regarded as an epiphenomenon associated with arrhythmia. However, recent landmark papers have revealed that cardiac leukocytes perform housekeeping functions essential for normal cardiac electrical conditions such as repolarization, acceleration, and recycling of cardiomyocyte-derived mitochondria in the steady state and in heart disease (8–13). These surprising, previously unknown, noncanonical functions of cardiac immune cells indicate that leukocytes themselves may be directly involved in the pathophysiology of arrhythmia. Hereinafter, we will discuss the role of neutrophil granulocytes (further referred to as neutrophils) in rhythm disorders and the potential mechanisms underlying neutrophil contribution to arrhythmia risk. For a broad overview of other immune cell populations such as macrophages, monocytes, or T/B cells and their role in arrhythmogenesis, we refer the interested reader to these excellent review articles (6, 14–16).

## MECHANISMS OF ARRHYTHMOGENESIS

Cardiac arrhythmias frequently affect the atria, for instance, in atrial tachycardia and atrial fibrillation (AF) (17, 18). Ventricular arrhythmias (VAs) include ventricular tachycardia, long-QT syndrome (LQTS), Torsades de Pointes, Brugada syndrome, and ventricular fibrillation (VF) (19). Conduction disturbances between atria and ventricles may result in atrioventricular (AV) node block or atrioventricular reentry (20). Despite the phenotypical diversity of cardiac arrhythmias, there are several common underlying electrophysiological mechanisms that can be divided into abnormal impulse formation and mechanisms related to abnormal impulse propagation (19).

### Abnormal Impulse Formation

Mechanistically, abnormal impulse formation (ectopic activity) is caused by abnormal automaticity or triggered activity resulting from early afterdepolarizations (EADs) or delayed afterdepolarizations (DADs) (21). Abnormal automaticity outside the sinus node is caused by continuous diastolic depolarization of the membrane potential until the threshold potential, where a new action potential (AP) is initiated. Abnormal automaticity is often mediated by a reduction in ion currents that stabilize the resting membrane potential (RMP; e.g., the basal inward-rectifier potassium current, *I*_K1_) or increased activity of hyperpolarization-activated pacemaker channels responsible for the funny current, I_f_ (17). Afterdepolarizations represent abnormal membrane depolarizations that occur during (EADs) or after (DADs) an AP. EADs typically occur at excessively prolonged APs, providing time for reactivation of l-type calcium channels (21, 22). DADs are ectopic diastolic depolarizations caused by abnormal Ca^2+^ release from internal Ca^2+^ stores (sarcoplasmic reticulum, SR) during diastole. The released Ca^2+^ is extruded from the cell by the Na^+^-Ca^2+^ exchanger (NCX) that creates a depolarizing inward current, which causes a short membrane depolarization (23, 24).

If large enough, EADs and DADs can trigger new APs and may thereby contribute to abnormal impulse formation and ectopic activity. Nevertheless, even subthreshold EADs or DADs may contribute to cardiac arrhythmias by causing severe conduction disturbances (25).

### Abnormal Impulse Propagation

Slowed electrical impulse propagation and heterogeneous conduction patterns have been shown to promote the occurrence of cardiac arrhythmias and both typically occur because of cardiac fibrosis. Advances in clinical imaging modalities have helped to establish the important role of fibrosis in arrhythmogenesis (26). In parallel, basic science has identified the mechanisms underlying the proliferation of fibroblasts and their differentiation into myofibroblasts, which is a central response to numerous stress signals activated in many cardiovascular diseases, resulting in excessive collagen production and fibrosis (27). Similarly, beat-to-beat variations in cardiomyocyte electrophysiology, called alternans, can lead to heterogeneities in signal propagation, thereby contributing to the development of arrhythmias (28–31).

### Reentry

Reentry describes an important paradigm in arrhythmia research (32). It describes the process of an excitation wave avoiding elimination, thereby causing a continuous ectopic activation of the surrounding myocardium. There are two main mechanistic concepts describing reentry: the leading-circle model and the spiral-wave model (33). The leading-circle model requires an unexcitable core, around which an excitation wave propagates. This propagation requires a suitable combination of conduction velocity and effective refractory period for the wave to avoid elimination by its own wave tail. In contrast, the spiral-wave model does not require an unexcitable core, but rather a functionally vulnerable substrate in which a stable spiral wave can form. The stability of this model is determined by the relationship between the tissue’s excitability and its ability to excite neighboring tissue areas (source-sink relationship), as well as the wave’s curvature (19).

## NEUTROPHIL GRANULOCYTES

Neutrophils are important protagonists of the innate immune system and represent 10–25% of all circulating leukocytes in mice and 50–70% in humans (34). Neutrophils in tissue or blood are terminally differentiated cells derived from immature neutrophils in the bone marrow. The mature, inactive neutrophil is released from the bone marrow into the circulation and is only activated upon priming in inflammatory conditions (e.g., ischemia, infection), causing extravasation into tissues via selectins, integrins, and chemokine receptors. Following migration into the tissue, neutrophils carry out various antimicrobial, proinflammatory effector functions such as degranulation, oxidative stress, or neutrophil extracellular trap (NET) formation. Of note, neutrophils are barely detected in the heart under physiological conditions (35, 36). When compared with other long-lived immune cells, neutrophils have a relatively short circulating half-life of only 6–12 h in mice and are quickly replenished by the release of large numbers from the bone marrow reservoir.

Only recently, neutrophil polarization into subsets with distinct functional identities has been revealed by novel multiparametric single-cell genomics profiling techniques (37–39). In line, mature neutrophil subsets in the circulation have been described in humans based on cell marker expression profiles (e.g., CD11c, CD62L, CD11b, CD16, CD177, olfactomedin-4) and have partly also been linked to distinct functionalities such as NET formation, T-cell inhibition, or angiogenesis in transplanted tissue. Temporal neutrophil polarization following ischemia indicates distinct neutrophil phenotypes within the postischemia time continuum (36). Importantly, the process of neutrophil polarization in cardiac diseases has been shown to start at the origin of emergency hematopoiesis in the bone marrow, far from the site of tissue injury, indicating previously unrecognized neutrophil diversity (37, 38). As of yet, it remains unknown if all neutrophil subpopulations and phenotypes contribute to arrhythmia risk in patients with underlying heart disease.

## ASSOCIATIONS BETWEEN NEUTROPHIL EXPANSION AND ARRHYTHMIA RISK

In this section, we will review the relationship between neutrophils and the development of cardiac arrhythmias in the context of various cardiovascular pathologies. The diseases discussed include myocardial infarction (MI), sepsis, myocarditis, and COVID-19.

### Myocardial Infarction

Myocardial infarction (MI) is caused by inadequate oxygen supply due to blocked coronary arteries as a result of ruptured or eroded arterial walls (40). As a consequence, ischemic injury develops downstream in the insufficiently oxygenated cardiac regions, resulting in myocyte death by apoptosis, necrosis, or pyroptosis (41–43). Arrhythmia risk and occurrence after MI is well known and has been reported by multiple clinical trials (44–46). AF, VF, and VT have been shown to occur most commonly after MI (47, 48). About 6% of patients that are admitted to the hospital with an acute MI develop VT or VF within 48-h posthospitalization (49). The occurrence of arrhythmias in the long term after MI reaches up to 46% in patients receiving an implantable cardiac monitor after the acute MI (50).

**Figure 1.**
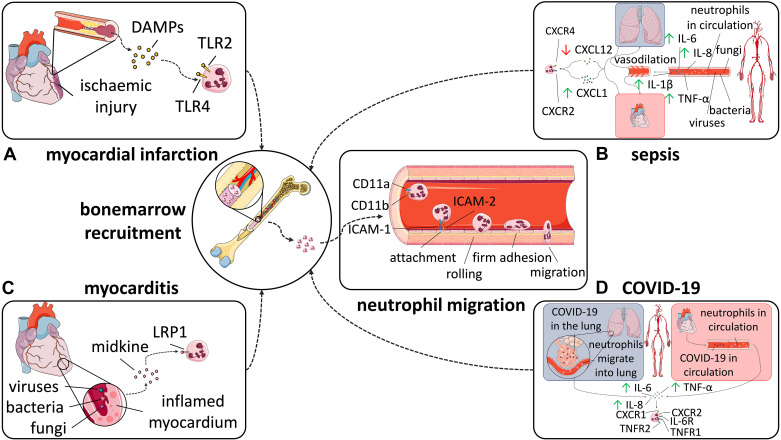
Conditions sharing neutrophil expansion and arrhythmia incidence. The occurrence of arrhythmias has been demonstrated in various different pathologies. *A*: myocardial infarction. Neutrophil spread in myocardial infarction is mediated by recognition of damage-associated molecular patterns (DAMPs) via Toll-like receptors 2 (TLR2) and 4 (TLR4), triggering neutrophil recruitment from the bone marrow to the site of cardiac injury. *B*: sepsis. Neutrophil proliferation in sepsis is driven by the presence of pathogens in the bloodstream that affects multiple organs, especially heart and lungs, causing upregulation of CXCL1 and downregulation of CXCL12, which ultimately promotes the recruitment of neutrophils from the bone marrow. *C*: myocarditis. Neutrophil expansion in myocarditis involves midkine-mediated promotion of neutrophil transport and NETosis in myocarditis, which is achieved through activation of lipoprotein receptor-related protein 1 on the surface of neutrophils, ultimately leading to increased cardiac inflammation and neutrophil recruitment from the bone marrow. *D*: COVID-19. Neutrophil expansion in COVID-19 is driven by COVID-19 pathogens in the bloodstream and lungs, resulting in increased levels of IL-6, IL-8, and TNF-α, which interact with their respective receptors on the neutrophil surface, including CXCR4, CXCR2, TNFR1, TNFR2, and IL-6R, promoting neutrophil activation and recruitment from the bone marrow to the site of infection. Parts of the figure were drawn by using pictures from Servier Medical Art. Servier Medical Art by Servier is licensed under a Creative Commons Attribution 3.0 Unported License.

In MI, damaged endothelial cells (ECs) and dying cardiomyocytes release damage-associated molecular patterns (DAMPs) that bind to pattern recognition receptors on neutrophils (Fig. 1*A*) (51). Neutrophils attach to ECs through the binding of intercellular adhesion molecule 1 (ICAM-1) and intercellular adhesion molecule 2 (ICAM-2) and integrins, such as CD11a and CD11b (8). Integrin binding results in a rolling effect of neutrophils against the vessel wall. When attached, neutrophils crawl along the endothelium following the chemotactic gradient until they find a suitable site for paracellular migration (52). Downstream, ICAM-integrin interactions facilitate the phosphorylation of vascular cadherin which, in turn, increases permeability of junctions in the inflamed endothelium (53). Neutrophil guidance across the vessel wall is directed by chemokine (CXC motif) ligand 1 and 2 (CXCL1 and CXCL2) (54). Although CXCL1 is produced by ECs stimulated with tumor necrosis factor (TNF), neutrophils themselves are the primary producers of CXCL2, conceptually leading to the paradigm whereby transmigrating neutrophils promote CXCL2-dependent, self-guided gradient-mediated migration through EC junctions (54).

Throughout neutrophil migration, Ca^2+^ regulates a wide range of neutrophil mechanisms including phagocytosis, migration, and degranulation (55). Ca^2+^ interacts with integrins and can induce and reverse adhesion (56). S100 calcium-binding protein A8 and A9 (S100A8 and S100A9) have been demonstrated to be released by neutrophils forming a S100A8/A9 heterodimer (known as calprotectin) that binds to Toll-like receptor 4 (TLR4) (57, 58). TLR4 signaling activates myeloid differentiation factor 88 (MyD88) and Toll/interleukin-1 receptor adapter proteins, which in turn triggers the recruitment of IL-1 receptor-associated kinases, TNFα receptor-associated factor 6, and a series of phosphorylation events (59), inducing downstream transcription of proinflammatory cytokines and subsequently amplifying tissue inflammation (60).

Of note, neutrophil signaling has been shown to be sex dependent in MI (61). Male animals tend to have stronger inflammatory responses in the early phase after MI compared with females, accompanied by higher levels of CXC motif chemokine receptor 3 (CXCR3), IL-6Rα, IL-13, and IL-1r1 (62). In line, female mice have better survival rates after MI of ∼80% compared with 60% in males (62). The sex-dependent differences post-MI may very likely be due to sex hormone distribution in males and females. Testosterone causes elevated peripheral neutrophil counts and enhances acute myocardial inflammation leading to impaired wound healing and remodeling, whereas estrogen was shown to enhance cardiomyocyte survival via inhibition of p53 activation and, hence, prevent cardiomyocyte apoptosis (63–65). These sex differences also apply to AF, where incidence rates per 1,000 person-yr was reported between 1.6 and 2.7 in women and between 3.8 and 4.7 in men, perhaps indicating a link between sex differences in neutrophil biology and arrhythmia burden (66).

Myocardial inflammation accompanying ischemic insult has previously been demonstrated to contribute to post-MI arrhythmia (67). This was proven by a landmark study demonstrating higher susceptibility to arrhythmia of MI mice with preexisting acute myocardial inflammation (67). Moreover, inhibition of IL-1β by Anakinra, acting as IL-1β receptor antagonist, improved conduction velocity post-MI, suggesting antiarrhythmic effects by blocking myocardial inflammation (68). In line, inhibition of IL-1β in type 2 diabetes mellitus mice rescued the susceptibility to VA, implying that cytokine abundance contributes to arrhythmia burden in MI (69). Moreover, we were able to demonstrate that neutrophils are causally involved in the pathogenesis of VAs early after MI by using the STORM (spontaneous tachycardia occurred frequently in hypokalemic mice with MI) model (9). Our work highlights neutrophil-mediated oxidative stress via lipocalin-2 (Lcn2) as a key mechanism for neutrophil’s proarrhythmogenic action (9). Using quantitative real-time PCR in sorted immune cells, we confirmed that post-MI neutrophils were associated with high expression of Lcn2 (9). In this study, the functional relevance of Lcn2 in ventricular arrhythmia was investigated by preparing Lcn2^+/+^ and Lcn2^−/−^ bone marrow chimeras resulting in bone marrow-derived cells, including neutrophils, lacking Lcn2. Although cardiac neutrophil counts were unaffected in Lcn2^−/−^ bone marrow chimeras after MI, ventricular arrhythmia burden was reduced in Lcn2^−/−^ bone marrow chimeras compared with Lcn2^+/+^ controls supporting the idea that Lcn2 contributes to arrhythmia burden in the ischemic myocardium (9). These findings are supported by studies highlighting the role of Lcn2 in MI and other cardiovascular conditions, yet the role of Lcn2 in ventricular arrhythmia is a novel concept (70, 71). Further research is warranted to elucidate exact pathomechanisms and the Lcn2-arrhythmia axis in MI and other rhythm disorders. Other mechanisms such as neutrophil-released cytokines were proposed to contribute to MI-associated arrhythmias, too. For instance, mice overexpressing TNF α, produced by neutrophils and other immune cells, were more susceptible to VAs (72, 73). Other proinflammatory mediators that link neutrophils to arrhythmia in the context of MI include IL-1β, IL-6, and IL-17A (74–76). Collectively, these data suggest a role for neutrophils in the pathogenesis of MI through migration, signaling, and proinflammatory effector functions, shaping the cardiac substrate and electrophysiology in ischemic hearts.

### Sepsis

Sepsis is defined as a syndrome of pathological and biochemical abnormalities induced by infection and is a leading cause of death and critical illness worldwide (77). Signs and symptoms of sepsis include tissue hypoperfusion, lactic acidosis, and oliguria, which in turn impact cardiac function and may cause arrhythmias (78–80). Previous studies have reported the development of supraventricular tachycardia (SVT) and AF in 6–20% of patients with severe sepsis (81–83).

Neutrophil biology has been widely reported as being heavily disturbed in sepsis, including impairment of housekeeping functions of neutrophils (Fig. 1*B*) (84). Sepsis causes impaired neutrophil migration and elevated release of neutrophils from the bone marrow, both contributing to increased neutrophil count in the circulation (85). Elevated levels of antiapoptotic protein myeloid leukemia cell differentiation protein (Mcl‐1) and antiapoptotic protein B-cell lymphoma-extra large (Bcl‐xL) extend neutrophil lifespan in sepsis (86, 87). In the bone marrow, CXCL12 downregulation and CXCL1 upregulation promote the release of neutrophils into the circulation (85). Expression of β_1_- and β_2_-integrins is upregulated in sepsis, resulting in decreased marginalization and deformability of neutrophils and sequestration in the capillary bed during the rolling phase (88), causing vascular obstruction, tissue ischemia, and organ dysfunction (88).

A link between neutrophils and arrhythmia in the context of sepsis has been demonstrated in several studies. For example, high levels of circulating histones, mainly derived from NETs, which are known to be elevated in patients with sepsis, were associated with the occurrence of SVTs, AF, and recurrent ectopic beats (89–91). In line, patients developing arrhythmia during sepsis had higher levels of C-reactive protein (CRP) (92, 93). As CRP is known to induce NETosis, diminished CRP levels may be a mechanism counterbalancing increased NET formation in sepsis (94). Moreover, sepsis patients with hyponatremia had a higher risk of developing arrhythmia (95, 96). Of note, hyponatremia and elevated neutrophil counts in systemic inflammation are directly correlated, supporting the idea that neutrophils contribute to arrhythmogenesis in sepsis (50). This is further supported by our recent demonstration that cecal ligation and puncture, a commonly used animal model of polymicrobial sepsis, can be used as a novel tool to mimic sepsis-induced AF in wild-type mice, emphasizing the interrelationship between sepsis and arrhythmia (97). Taken together, cumulative evidence points toward neutrophil involvement in the pathogenesis of arrhythmia-associated sepsis. However, direct evidence causally linking neutrophils to arrhythmogenesis in sepsis is still lacking.

### Myocarditis

Myocarditis is an inflammatory disorder caused by viruses, bacteria, or fungi, toxic substances or systemic diseases such as systemic lupus erythematosus, sarcoidosis or dermato-polymyositis (Fig. 1*C*) (98). Among the most common symptoms, life-threatening VAs have been shown to manifest in the context of myocarditis (99). Of 185 patients admitted with myocarditis and VAs consecutively, malignant VAs occurred in 55 (30%) during a prospective follow-up period of 27 ± 7 mo (100). Given that arrhythmias are usually caused by either triggered activity or reentry, there are various potential mechanisms underlying the relationship between myocarditis and arrhythmia (101). These include myocardial replacement fibrosis, promoting reentry mechanisms, altered Ca^2+^ handling, modified gap junction function, and proarrhythmic effects due to the release of cytokines (102).

Neutrophil counts peak 7 days postinfection, and inhibition of neutrophils in the acute phase of infection improves cardiac inflammation in a mouse model of myocarditis (103). Increased oxidative stress, a key feature of myocarditis, induces the release of TNF-α and IL-6, both known to be secreted by neutrophils and contributing to electrical instability (104). There are certainly descriptive studies linking myocarditis to increased arrhythmia burden and cardiac neutrophil expansion, although mechanistic involvement of neutrophils in myocarditis-associated arrhythmogenesis is not yet fully understood.

### COVID-19

COVID-19, also known as severe acute respiratory syndrome coronavirus type 2-SARS-CoV-2, has already affected roughly 770 million people to this date, causing ∼7 million deaths according to the World Health Organization (WHO) (covid19.who.int; accessed 19 September, 2023). COVID-19 is associated with increased abundance of inflammatory transcripts and in severe cases accompanied by multiorgan failure and cardiovascular disorders (105, 106). Clinical data from 4,526 patients with COVID-19 followed over 6 mo revealed an overall arrhythmia incidence of ∼20%, including sinus tachycardia, AF, VT, and VF (107, 108). Moreover, patients with COVID-19 often experience palpitations after recovering from acute illness caused by the virus, raising concerns regarding the potential proarrhythmic effects of the virus itself (109).

Severe COVID-19 cases have been linked to elevated neutrophil-to-lymphocyte ratios and increased serum levels of neutrophil-associated cytokines such as IL-6 (Fig. 1*D*) (110). In line with this, elevated IL-6 serum levels have been demonstrated as a predictor for the development of fatal pneumonia in patients with COVID-19, but are also associated with QT prolongation and persistent atrial inflammation, potentially leading to increased arrhythmia risk in COVID-19 (108, 111, 112). Beyond cytokine release by neutrophils, NET formation has been observed to be dysregulated in severe COVID-19 (110). Elevated levels of free DNA and citrullinated histone H3 were observed in sera of patients with COVID-19 compared with serum samples from healthy controls (113). As postulated earlier, increased NET formation can itself result in localized myocardial ischemia, cell death, and microvascular rarefaction, all associated with cardiac hypertrophy (114). Cardiac hypertrophy affects the heart’s conduction system, hence representing a secondary risk factor for arrhythmia in COVID-19 (115, 116).

Lastly, the NLR family pyrin domain containing-3 (NLRP3) inflammasome and its downstream molecular signatures, caspase-1 subunit p20 and IL-18, were measured in cardiac tissue from patients with COVID-19 postmortem, revealing that activation of the NLRP3 inflammasome associates AF and patient outcome (117, 118). Mechanistic evidence establishing a causal link between COVID-19 and cardiac arrhythmias is still missing, which is, at least in part, caused by the lack of available mouse models. Because COVID-19 has only recently become a research topic of interest, the possibilities to investigate the associated mechanisms leading to arrhythmias are far from exhausted.

## GENERAL CONCEPTS FOR CELLULAR INTERACTION BETWEEN CARDIOMYOCYTES AND NONMYOCYTES

**Figure 2.**
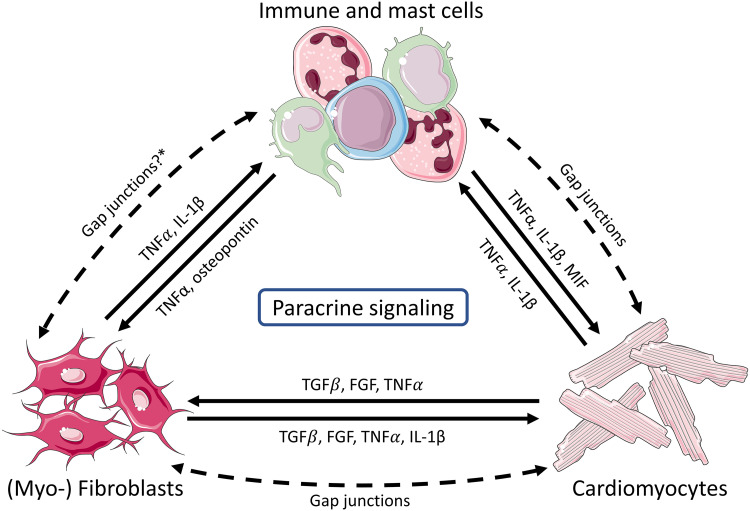
Interactions of cardiomyocytes with (myo-)fibroblast, immune cells, and mast cells. Major cytokines involved in paracrine signaling are summarized. (Myo-)fibroblasts and cardiomyocytes can interact through secretion of transforming growth factor β (TGFβ), fibroblast growth factor (FGF), tumor necrosis factor α (TNFα), and IL-1β. Immune cells can affect the other cell types by secreting TNFα, IL-1β, macrophage migration inhibitory factor (MIF), and osteopontin. Lastly, cardiomyocytes and (myo-)fibroblasts can modulate the activity of immune cells by secreting TNFα and IL-1β. In addition, cardiomyocytes can form gap junctions with noncardiomyocytes, allowing these cells to passively affect action potential propagation. *Gap junction formation between (myo-)fibroblasts and immune cells such as macrophages has not been shown but might enable a direct interaction. Parts of the figure were drawn by using pictures from Servier Medical Art. Servier Medical Art by Servier is licensed under a Creative Commons Attribution 3.0 Unported License.

Although the role of cardiomyocytes and fibroblasts in the development of cardiac arrhythmias has been studied extensively in recent years (26, 27, 119), the role of immune cells has generally attracted less attention (16). However, a few landmark studies have drawn attention to the role of immune cells in cardiac arrhythmogenesis over the past 5 years (9–11). Three potential underlying mechanisms are summarized in Fig. 2.

### Direct Cellular Coupling

Gap-junction formation between cardiomyocytes and noncardiomyocytes such as fibroblasts, myofibroblasts, and macrophages, further referred to as heterocellular coupling, has been recognized over the past decade (10, 120, 121). Although myofibroblasts or macrophages are nonexcitable, they can passively regulate the AP shape and excitation propagation, and may thereby impact (electro-)physiology of cardiomyocytes (16, 119, 122). Moreover, resident macrophages have been shown to form gap junctions with cardiomyocytes particularly in the AV node, thereby affecting resting membrane potential and refractoriness of adjacent cardiomyocytes, as well as passively contributing to signal propagation (10).

Neutrophils coupling to macrophages have been previously observed in the bone marrow. The functional relevance of neutrophil-macrophage heterocellular coupling is currently not known (123, 124). In MI, neutrophil-macrophage cross talk relies, at least in part, on secretion of cytokines and chemokines (125). Neutrophils release mediators through secretory granules that specifically attract circulatory proinflammatory Ly6C^hi^ monocytes to the ischemic myocardium, which shape the inflammatory milieu post-MI (125, 126). Moreover, neutrophils mediate a shift from proinflammatory macrophages toward a reparative phenotype that is involved in clearing dead neutrophils and cellular debris in the infarcted heart (123, 127). Concluding, neutrophils do cross talk with macrophages through indirect mechanisms. Whether direct heterocellular coupling between neutrophils and macrophages occurs in the heart in steady state or after MI is not yet conclusively investigated.

### Mediators Secreted by Immune Cells

Paracrine activity is a central aspect of immune cell communication. Hence, paracrine factors may play a central role in heart diseases associated with activation of the immune system (128–130). Apart from their effector functions in immune cell signaling, paracrine factors released by immune cells and other sources (see below) may considerably affect cardiomyocyte function. Cytokine receptor expression has been demonstrated in cardiomyocytes, rendering the cardiac AP vulnerable to inflammatory mediators (131, 132).

### Mediators Secreted by Cardiomyocytes or Fibroblasts

Activated or injured cardiomyocytes secrete factors that activate fibroblasts by promoting the transition into myofibroblasts and increasing the secretion of matricellular proteins (133). In turn, myofibroblasts regulate cardiomyocyte morphology and function by secreting factors such as transforming growth factor β (TGFβ) and fibroblast growth factor (FGF) (134–137). Cumulative evidence suggests that cardiomyocytes and fibroblasts may also regulate activity of immune cells in a paracrine manner, adding complexity to heterocellular interaction in the heart. For a more complete overview of paracrine interactions, we refer the reader to recent excellent reviews (131, 135, 138).

## NEUTROPHIL-SPECIFIC MECHANISMS IMPACTING ARRHYTHMIA

**Figure 3.**
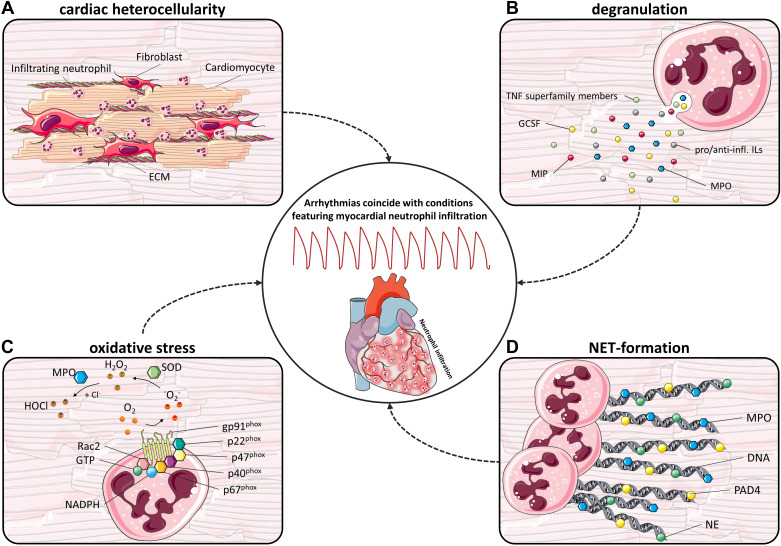
Neutrophil involvement in the onset of cardiac arrhythmias. Arrhythmias have been linked to a number of neutrophil-mediated host defense mechanisms that are explained in the following. *A*: cardiac heterocellularity. Neutrophils invade the myocardium in great numbers and are thought to significantly contribute to increased heterocellularity that likely impairs proper cardiac conduction. ECM, extracellular matrix. *B*: neutrophil-mediated modulation of the inflammatory response by degranulation. Neutrophils are producers of a wide range of pro- and anti-inflammatory mediators [among them tumor necrosis factor (TNF) superfamily members, granulocyte colony-stimulating factor (GCSF), macrophage inhibitory peptide (MIP), MPO, as well as a ladder of pro- and anti-inflammatory interleukins (ILs)]. *C*: oxidative stress. Neutrophil activation leads to assembly of the nicotinamide adenine dinucleotide phosphate (NADPH) oxidase (NOX) complex consisting of the catalytic core (gp91*^phox^* and p22*^phox^*), its subunits (p47*^phox^*, p40*^phox^*, p67*^phox^*), as well as Rac2 in its GTP-bound form. Upon NADPH binding, it cleaves the hydrogen and transports it to the extracellular space where a superoxide anion radical (O_2_^•−^) is generated. The superoxide dismutase (SOD) then generates hydrogen peroxide (H_2_O_2_), which subsequently is a substrate for myeloperoxidase (MPO)-mediated hypochlorous acid (HOCl) synthesis. *D*: neutrophil extracellular trap (NET) formation. As part of the neutrophil-mediated host defense, neutrophils evert nuclear and mitochondrial DNA fragments armored with antimicrobial proteins such as MPO, neutrophil elastase (NE), or protein arginine deiminase 4 (PAD4) to “trap” and neutralize invading pathogens. This process can either go along with neutrophil death, termed NETosis, or occur as vital NET-formation. Parts of the figure were drawn by using pictures from Servier Medical Art. Servier Medical Art by Servier is licensed under a Creative Commons Attribution 3.0 Unported License.

The incidence of arrhythmia in conditions featuring neutrophil expansion is suspected to be not merely coincidental, but rather due to a causal association leading to the onset of arrhythmic events in the injured heart, following the previously cited concept of electroimmunology. This section describes the various mechanisms that have been proposed and/or proven to drive neutrophil-mediated development of arrhythmia (Fig. 3).

### Heterocellularity and Cardiac Conduction

The heart, as centerpiece of the body’s circulation, is a complex organ comprising numerous cell types. Unsurprisingly, cardiomyocytes make up 90% of the cardiac mass (139) while representing ∼30% of atrial cells and 50% of ventricular cells (140, 141). The second most abundant cell type is the cardiac fibroblast, accounting for ∼25% of atrial and 15% of ventricular cells (141). They are vital for providing physical tissue stability by producing extracellular matrix components (142). Other common cell types are part of the cardiac vasculature, including smooth muscle cells, pericytes, and ECs (143), as well as adipocytes, mesothelial, and neuronal cells (141). Interestingly, cells of the innate immune system are also present in considerable numbers, with macrophages the most abundant population, representing 60–70% of all immune cells in the heart and 5–10% of all cardiac cells, being present in larger numbers in the atria than the ventricles (141, 144).

Cardiac electrical conduction is a tightly regulated process that follows a specific route and facilitates synchronized contraction of the heart. Starting out at sinoatrial node, conduction is propagated via the atrioventricular node, His bundle, left/right bundle branch, and the Purkinje fibers (145). On a cellular level, conduction between myocytes is usually facilitated via connexin 43 (Cx43)-containing gap junctions (146). Gap-junctional remodeling is present in numerous pathologies and can cause aberrant conduction and facilitate arrhythmia onset. For example, Cx43 expression is reduced post-MI, contributing to slowing of cardiac conduction. Interestingly, *Cx43* gene transfer or engraftment of *Cx43*-expressing cells reduces the susceptibility to ventricular tachycardia (VT) in different animal models, providing an important proof of concept for the antiarrhythmic effects of targeting the mechanisms underlying slow conduction (147–149). Finally, connexin hemichannels have also previously been shown to contribute to depolarizing inward currents that may facilitate the occurrence of cardiac arrhythmias (28, 150).

Although the concept of structural coupling by gap junctions has been applied to homocellular coupling exclusively, newer evidence suggests the possibility of cardiac heterocellular coupling between cardiomyocytes and noncardiomyocytes in the steady state (151). This has been extensively described for cardiac fibroblasts, expressing voltage-sensitive ion channels and also Cx43 (120, 152, 153). Interestingly, a recent study has elucidated a conductional involvement of cardiac macrophages in the setting of heterocellular coupling, expressing Cx43 and being coupled to cardiomyocytes, having a negative resting membrane potential and depolarizing in tandem with cardiomyocytes (10). Neutrophils have been also shown to form gap junctions, yet only described for coupling with endothelial cells, here regulating transmigration, and also to other neutrophils, while direct interactions with cardiomyocytes have not been observed so far (154, 155).

Many cardiac pathologies involve complex remodeling of the cardiac substrate by modification of the cellular composition, functionally disturbing cardiac conduction (156). For example, cardiac fibrosis with the proliferation of cardiac fibroblasts and increased deposition of matricellular proteins has been linked to AP prolongation and increased risk for the development of EADs or DADs (157). Neutrophil recruitment in large numbers to the diseased heart is a common feature of systemic inflammatory diseases like severe COVID-19 (158) and sepsis (159) but is also present in local ischemic cardiac injury (160). Data from the novel STORM mouse model demonstrate that ∼4,000 neutrophils per mg heart tissue migrate early after MI, shaping focal hotspots around the coronary circulation as neutrophils egress from the circulation into the cardiac periphery (9). These hotspots are not confined to the ischemic area and border zone but also spread through the entire myocardium, forming patchy areas of cellular and electrical heterogeneity of the cardiac substrate. In STORM mice, the presence of neutrophils was directly associated with the VA burden, as depletion of circulatory neutrophils could ameliorate the VA burden (9, 161). Clinical data match these findings, showing a correlation between circulating neutrophil count and the occurrence of arrhythmic events and cardiac arrest (9, 161). Based on this evidence it seems likely that the sudden emergence of otherwise absent neutrophils in the injured heart and the resulting pathological heterocellularity impacts physiological electrical conduction through the myocardium (Fig. 3*A*) (162–164). As conditions like myocarditis or MI feature both extracellular matrix deposition and immune cell recruitment, it remains challenging to distinguish to what extent the individual mechanisms and cell populations contribute to overall arrhythmogenicity (165).

### Release of Proinflammatory Mediators

Neutrophils are primarily associated with their phagocytic and antimicrobial abilities, yet have also been shown to mediate local inflammatory responses, e.g., after MI or myocarditis, when reaching their peripheral target site and becoming increasingly transcriptionally active (43, 103, 166, 167). Although the total protein content synthesized by individual neutrophils may seem low, the immense quantity of neutrophils makes them a decisive contributor toward the inflammatory fate when entering the site of inflammation as first responders of the immune system (168, 169). With this in mind, it is not surprising that neutrophils are capable of producing a vast range of chemokines that not only orchestrate recruitment of monocytes, dendritic cells, natural killer cells, and T cells but also fuel further neutrophil recruitment (73, 170, 171). This is commonly mediated by infections, activating neutrophil surface receptors, or via pathogen-associated molecular patterns (PAMPs) or DAMPs (51, 172). Release of proinflammatory cytokines through degranulation further fuels the inflammatory milieu and involves IL-1α, IL-1β, IL-6, IL-17, IL-18, IL-22, macrophage migration inhibitory factor (MIF), granulocyte colony-stimulating factor (GCSF), and members of the TNF superfamily (Fig. 3*B*) (73, 173–176). However, it should also be noted that neutrophils play an important role in resolving inflammation, through their release of anti-inflammatory mediators such as IL-10 and TGFβ (73, 177, 178).

In the past, inflammation has been regarded as an epiphenomenon in diseases associated with arrhythmia onset. Only recently, mounting evidence establishes a direct link between arrhythmia and the release of proinflammatory cytokines in the heart (14, 179). Mechanistically, arrhythmogenesis is primarily mediated by mutations, altered expression, composition, trafficking, and function of ion channels, resulting in abnormal ion currents with subsequent effects on the tightly regulated cardiac AP (180–182). In rodent experiments, increased abundance of TNF-α was associated with reduced transient outward K^+^ currents and subsequent AP and QT prolongation (72, 183–185). Similar effects were reported for high IL-1β levels in diabetic mice (186). IL-6 and IL-17 were shown to directly affect ion currents, the former affecting l-type Ca^+^ channel influx and slow and rapid K^+^ rectifier currents, the latter decreasing transient outward K^+^ currents, both causing AP and QT prolongation (14). Finally, cytokines may affect intracellular Ca^2+^ handling. IL-1β, for example, has been shown to promote occurrence of spontaneous Ca^2+^ releases from the sarcoplasmic reticulum and thereby trigger AF episodes following cardiac surgery (28, 132). In addition to direct effects on cardiac electrophysiology, systemic effects of proinflammatory cytokine release must be considered. QT prolongation has been found in a great range of systemic inflammatory diseases such as rheumatoid arthritis, systemic lupus erythematosus, or connective tissue disease (187–190). In addition, fever, cytochrome P450 inhibition, and aromatase stimulation have been discussed as potential contributors to AP prolongation(112).

In summary, neutrophils substantially contribute to inflammatory cytokine release in systemic (auto-)inflammatory diseases, which causes AP alterations and primarily QT prolongation, resulting in increased risk for life-threatening arrhythmic events such as Torsades de Pointes.

### Oxidative Burst

In infectious diseases, proper activation of the innate immune system including neutrophil expansion is a key feature of host defense. A well-known neutrophil response to pathogens is the synthesis of superoxide anions and downstream products, referred to as reactive oxygen species (ROS), directed to kill pathogens in internalized phagosomes or the interstitial space via degranulation of antimicrobial proteins (Fig. 3*C*) (191). This process, termed oxidative burst, can occur during infection and sterile inflammation and is specific to neutrophils and other phagocytes (192, 193). It is regulated via neutrophil surface receptors that bind pathogenic peptides or proinflammatory cytokines and trigger intracellular signaling cascades (193–195). To name only a few, G protein-coupled receptors (GPCRs), Fc receptors, adhesion molecules, cytokine receptors, and TLRs expressed on neutrophils (194), all of which directly or indirectly facilitate ROS production upon activation through binding of bacterial proteins or inflammatory mediators (196). Downstream, receptor activation triggers the assembly of the nicotinamide adenine dinucleotide phosphate (NADPH) oxidase (NOX), a central player in ROS formation in neutrophils as it generates superoxide anions (197, 198). In the steady state, the NOX catalytic core unit (also termed flavocytochrome b558, short cytb_558_), consisting of gp91^phox^ and p22^phox^, resides in the phagosome, secretory vesicles, or cell membrane. The regulatory subunits (p47^phox^, p67^phox^ and p40^phox^) of the NOX complex reside within the cytosol among the Ras-related C3 botulinum toxin substrate 2 (Rac2) GTPase, which is additionally required for NOX activation (196, 199). Upon NOX activation, phosphorylation of its subunits allows for translocation and binding of the cytosolic components to cytb_558_ alongside binding of Rac2 in its GTP-bound form (191, 200). NOX activity has additional regulatory layers that are described in greater detail elsewhere (201–204). The activated NOX complex will then be transported to the neutrophil’s membrane to shuttle electrons from intracellular NADPH to extracellular or phagosomal oxygen, thereby generating superoxide anions which can in turn be converted into hypochlorous acid, hydrogen peroxide, or others (205). This conversion is, among other enzymes, mediated by the heme-enzyme myeloperoxidase (MPO) and the superoxide dismutase (SOD) (206). In addition to ROS’ microbicidal features, they can also act as a second messenger in the heart, mediating inflammation and further ROS production in the surrounding tissue, also known as ROS-induced ROS release (207). In particular, further ROS production by cardiomyocytes should be noted, which has been described for a number of cardiac pathologies such as ischemia-reperfusion injury, heart failure, and diabetic cardiomyopathy (208). Mitochondria represent ∼30% of the cardiomyocyte’s volume and are hence also a major ROS-producing entity in the heart under physiological and pathophysiological circumstances (209, 210). Dysregulation of the mitochondrial respiratory chain has been previously shown to be directly linked to increased ROS levels in the failing myocardium (209). Interestingly, cardiomyocytes have been shown to express NOX isoforms, just like neutrophils, that are responsible for ROS production in the failing myocardium (211).

A number of studies have previously highlighted a direct link between increased ROS production and the onset of arrhythmias in the ischemic/postischemic heart, either mediated via neutrophils and their release of the defense protein Lcn2 or by alterations in mitochondrial membrane potentials that directly affect the tightly regulated AP (9, 212, 213). Specifically, oxidative stress has been directly linked to altered ion channel expression patterns and function, disturbing the tightly regulated cardiac AP (210). Myocytes exposed to H_2_O_2_ demonstrated lower expression of the Na^+^ voltage-gated channel α-subunit 5 (SCN5A) and subsequently reduced inward Na^+^ currents, jeopardizing proper depolarization and AP propagation. In line, cardiomyocyte’s Ca^2+^ handling is impaired in conditions of increased oxidative stress, which acts as arrhythmic substrate (214). Furthermore, key components of cardiac Ca^2+^ handling are impacted by oxidative stress: *1*) RyR2, responsible for the Ca^2+^-induced Ca^2+^ release, undergoes sulfhydryl group oxidation, leading to reduced Ca^2+^ transients and Ca^2+^ leak from the SR (215, 216), *2*) the SR Ca^2+^ ATPase SERCA, actively transporting Ca^2+^ into the SR, likewise undergoes sulfhydryl group oxidation upon increased oxidative stress, leading to prolongation of the AP (217, 218), and *3*) the NCX, shuttling Ca^2+^ outside of the cell during diastole, becomes stimulated and is associated with DADs (219, 220). Ca^2+^/Calmodulin-dependent protein kinase a major regulator of Ca^2+^-dependent signal transduction, is regulated by oxidation and contributes to various forms of cardiac arrhythmias such as AF (18, 221–223). Lastly, K^+^ channels are also affected by increased ROS production, evident as decreased abundancy of the human ether-à-go-go related gene (hERG) K^+^ channel, as well as altered activity, increasing the likelihood for ectopic activity (224, 225). Increased ROS production was associated with loss of the gap junctional protein Cx43, severely affecting electrical coupling of cardiomyocytes and leading to a functionally impaired syncytium with high arrhythmogenic potential (226).

Taken together, the current literature provides ample evidence for the role of neutrophil-associated ROS production, which is able to increase arrhyhmogenicity by various mechanisms.

### Neutrophil Extracellular Trap Formation

Besides neutrophil’s antimicrobial abilities mediated by the release of enzymatic granule content or phagocytosis of pathogens, an additional mechanism of neutrophil-mediated host defense is NET formation (227). NETs are extracellular DNA webs armed with antimicrobial proteins that can trap and directly eliminate pathogens (Fig. 3*D*) (228, 229). Increased NET formation has been observed in not only patients with AF and MI but also preclinical settings of myocarditis (230–232). The presence of NETs is associated with NET-dependent neutrophil death termed NETosis, as the neutrophil everts its nuclear and mitochondrial DNA (233). However, there are also reports about neutrophils being able to survive and thrive alongside NET formation, termed vital NET formation or vital NETosis (234, 235). Central to the onset of NETosis is the release of proteins from intracellular granules, among them neutrophilic elastase (NE) and the heme-enzyme MPO (233). NE, a serine protease, contributes to cytoskeletal reorganization and degradation, which is required for NETosis while MPO is a key mediator of granular release without the need for MPO enzymatic activity (236). The peptidyl-arginine deaminase 4 (PAD4) is responsible for hypercitrullination of histones and is involved in chromatin decondensation in neutrophils (237). Its inhibition results in the absence of NETosis and loss of NET-mediated pathogen death (238).

In infectious conditions, NET formation is centrally important as a host-defense mechanism while overshooting NETosis can also be harmful, especially in the heart and in ischemia-associated sterile inflammation (239). This is reflected by numerous studies investigating the beneficial effects of NETosis inhibition, e.g., by targeting MPO or PAD4 (239). As outlined earlier, neutrophil-derived MPO is involved in the production of ROS, mediating NET formation, and after release in the extracellular space amplifies local immune responses (233, 236). Elevated MPO levels have been directly linked to cardiac remodeling and AF onset in patients (240, 241). Moreover, MPO deficiency in mice was associated with beneficial effects, yielding protection from AF upon right-atrial stimulation (240). MPO also mediates postischemic remodeling in the heart and thereby indirectly contributes to arrhythmogenicity (242). However, MPO-mediated arrhythmogenicity may not be specifically and solely linked to NET formation but rather a combined result of ROS- and NET-mediated effects. In contrast, PAD4 inhibition, and therefore inhibition of NET formation, reduced infarct size, rescued cardiac function, and ameliorated neutrophil recruitment and NET formation in MI, all factors that lower the likelihood for arrhythmia onset (243). As of yet, there is no direct line of evidence that links PAD4 to any type of arrhythmia. Although, in the light of the beneficial effects of MPO inhibition, it seems reasonable to speculate that inhibition of other neutrophilic proteins such as PAD4 has similar antiarrhythmic effects in the MI setting, as it reduces inflammation and infarct size, both parameters known to directly correlate with arrhythmia burden in patients (244, 245). On the other hand, neutrophils are not the only source of PAD4 in the human body and PAD4 is also not only involved in NETosis but also other physiological processes (246). Therefore, experiments with neutrophil-specific PAD4 knockout or knockdown are warranted to further elucidate the effects of neutrophil-derived PAD4.

Overall, NET formation is a common mechanism in (sterile) inflammation and coincides with the onset of various arrhythmia types in preclinical and clinical studies.

## CHALLENGES AND POTENTIAL OF NEUTROPHIL-SPECIFIC THERAPIES

The relevance of neutrophil-mediated tissue damage in inflammatory conditions has been known for decades and with this also the idea of developing neutrophil-based targeted therapies (247, 248). Regarding MI, we and others have shown previously that the numbers of circulating neutrophils after MI are elevated in patients and were correlated with lower survival (9, 249, 250). In this instance, the idea of antineutrophil treatment would not only aim at reducing tissue damage, scar size, and preserving myocardial function but also to inhibit direct neutrophil actions as oxidative stress to reduce arrhythmia burden after MI.

However, total neutrophil ablation in patients in intensive care unit (ICU) seems a high-risk endeavor, as this strategy comes with the costs of a compromised innate immune system, resulting in increased vulnerability to potential infections (251). Moreover, neutrophils are centrally involved in inflammation resolution and scar maturation after MI (252–254). Neutrophil-ablated mice showed higher apoptotic cell counts after MI compared with the controls (123). In light of these thoughts, it has to be acknowledged that broad and sustained neutrophil ablation may have adverse effects on scar formation or infection risk. Targeting rather neutrophil subsets or proteins (e.g., Lcn2) specifically, to prevent overshooting inflammatory responses, seems to be preferable. Yet, general consensus on defined neutrophil subsets and their dynamics after MI does not exist, hindering the development of specific therapeutics. Further research on the characteristics of neutrophil subsets in distinct sterile inflammatory conditions is warranted to come to a general agreement on homeostatic and proinflammatory subsets and their functions.

## CONCLUSIONS

Neutrophils are central protagonists of cardiac inflammation during heart disease and their large quantity and diverse effector functions shape the arrhythmogenic substrate in multiple facets, in line with the concept of electroimmunology. As of yet, the role of neutrophils in rhythm disorders is only emerging as direct evidence proving causality is sparse, at least in part, due to a lack of neutrophil-specific target options (e.g., LysM-Cre or neutrophil-depleting antibodies). Recent recognition of neutrophil diversity challenges some long-held preconceptions about neutrophils and adds an additional layer of complexity to the already complicated inflammatory cascade in heart disease and secondary arrhythmia outcomes. Dissecting the role of neutrophil effector functions in the context of underlying heart disease will be crucial in improving our understanding of neutrophil’s role in arrhythmogenesis and ultimately facilitating the development of tailored therapies.

## GRANTS

J.G. is supported by the German Centre for Cardiovascular Research (DZHK), German Research Foundation (DFG) Grants CRC-1470 A04, CRC-1425, and 422681845, DynAge Freie Universität (FU) Berlin, the German Society for Cardiology (DGK), Corona-Stiftung, dt. Stiftung für Herzforschung and Charité 3 R. N.V. is supported by the Deutsche Forschungsgemeinschaft (DFG) Grants VO 1568/3-1, VO 1568/4-1, SFB1002 project A13, and under Germany’s Excellence Strategy – EXC 2067/1–390729940), by the DZHK Grants 81X4300120 and 81X4300102 (“DNAfix”).

## DISCLOSURES

No conflicts of interest, financial or otherwise, are declared by the authors.

## AUTHOR CONTRIBUTIONS

J.G. conceived and designed research; N.H., L.B., Y.D., and N.V. prepared figures; N.H., L.B., Y.D., N.V., and J.G. drafted manuscript; N.H., L.B., Y.D., N.V., and J.G. edited and revised manuscript; N.H., L.B., Y.D., N.V., and J.G. approved final version of manuscript.
